# Clinical and demographic profile of users of a mental health system for medical residents and other health professionals undergoing training at the Universidade Federal de São Paulo

**DOI:** 10.1590/S1516-31802004000400004

**Published:** 2004-07-01

**Authors:** Rafael Fagnani, Cristina Sueko Obara, Paula Costa Mosca Macedo, Vanessa Albuquerque Cítero, Luiz Antonio Nogueira-Martins

**Keywords:** Health services, Internship and residency, Graduate education, Professional burnout, Occupational health. Mental Health, Serviços de saúde, Residência médica, Educação de pós-graduação, Estafa profissional, Saúde ocupacional, Saúde mental

## Abstract

**CONTEXT:**

A postgraduate and resident trainee mental health assistance center was created in September 1996 within our university.

**OBJECTIVE:**

To describe the clinical and demographic profile of its users.

**TYPE OF STUDY:**

Retrospective.

**SETTING:**

Universidade Federal de São Paulo — Escola Paulista de Medicina (Unifesp-EPM).

**METHODS:**

The study was carried between September 1996 and November 2002, when 233 semi-structured registration forms were filled out either by the psychologist or the psychiatrist during their first contact with the trainees, who were medical and nursing residents, and postgraduate students at specialization, master or doctoral levels. The registration forms included demographic, occupational and clinical data.

**RESULTS:**

The trainees were predominantly young (mean of 27 years old), single (82.0% of cases), women (79.4%), seeking help especially during the first year of training (63.1%). In 70.8% of the cases, they came to the service spontaneously. Such individuals showed greater adherence to the treatment than those who were referred by supervisors (p < 0.05). In 30% of the cases, the trainee sought psychological guidance or support at the service due to specific situational conflicts. Depression and anxiety disorders were the most frequent diagnoses; 22.3% of the trainees followed up mentioned a tendency towards suicidal thoughts. In comparison with other trainees, there was a higher prevalence of males among the medical residents (p < 0.01), with more cases of sleep disorders (p < 0.05), a smaller number of individuals refraining from the use of alcohol (p < 0.05) and a higher number of trainees requiring leave of absence (p < 0.001).

**DISCUSSION:**

The first year of training in health sciences is the most stressful, especially for women. Depression and anxiety symptoms are common, reflecting transitory self-limited deadaptation. However, the severity of the cases can also be evaluated in view of the large number of trainees who mentioned suicidal tendencies.

**CONCLUSIONS:**

This study emphasizes the need and importance of providing formal, structured and confidential mental health services for medical residents and postgraduate students from other health professions, in the training programs of academic institutions.

## RESUMO

**CONTEXTO:**

O serviço Napreme foi criado em setembro de 1996 dentro de nossa universidade.

**OBJETIVO:**

Descrever o perfil clínico e demo-gráfico dos usuários.

**TIPO DE ESTUDO:**

Retrospectivo.

**MÉTODOS:**

Estudo de 233 registros semi-estruturados, preenchidos pelo psicólogo/psiquiatra, durante o primeiro atendimento do usuário (médico ou enfermeiro residente, pós-graduando ou especializando) no período de 01/12/1996 a 30/11/2002. O registro continha dados sócio-demográficos, ocupacionais e clínicos do usuário do serviço.

**RESULTADOS:**

Os usuários tinham idade média de 27 anos, predomínio do sexo feminino (79,4%), eram solteiros (82,0%), e procuravam ajuda no primeiro ano de treinamento (63,1%). Em 70,8% dos casos, a procura foi espontânea, sendo a adesão ao tratamento maior nesse grupo em comparação aos usuários que foram encaminhados por supervisores (p < 0,05). Em 30% dos casos, o usuário procurou o serviço em busca de orientação psicológica ou suporte com relação a conflitos situacionais específicos. Transtornos depressivos e ansiosos foram os diagnósticos mais freqüentes; 22,3% dos usuários atendidos referiram ideação suicida. Os médicos-residentes diferiram do restante dos usuários por serem predominantemente do sexo masculino (p < 0,01), com mais distúrbios do sono (p < 0,05), com menor número de abstêmios (p < 0,05) e por necessitarem de mais licenças e afastamentos (p < 0,001).

**DISCUSSÃO:**

Em conformidade com a literatura, o primeiro ano de treinamento é mais estressante, sobretudo para as mulheres. A maior parte dos sintomas apresentados é do espectro depressivo-ansioso, refletindo transtornos de adaptação. No entanto, a gravidade pode ser avaliada tendo-se como critério o grande número de casos em que tendências suicidas foram referidas.

**CONCLUSÕES:**

Este estudo enfatiza a necessidade e a importância de se prover serviços formais, estruturados e confidenciais de assistência à saúde mental para médicos residentes e pós-graduandos da área da saúde.

## INTRODUCTION

A high prevalence of suicides, depression, psychoactive substance abuse, stress, impairment, burnout and professional dysfunction among physicians, as well as the especially high rates of stress and depression among residents, have been described in the literature.^1-17^ With regard to professional dysfunction, it is worthwhile emphasizing the importance of recent studies referring to “problem residents”.^18,19^ Physicians’ fatigue in relation to patient safety,^20^ as well as residents’ weekly workload,^21^ have once again been brought up for discussion, and have now become a matter of interest.

Over the last twenty years, several authors have presented different suggestions for the prevention and management of personal and professional problems occurring during medical residence training programs.^22-24^ Consequently, the Universidade Federal de São Paulo (Unifesp) advocates that stress reduction during the training program — thereby contributing towards personal and professional growth, and preventing professional dysfunction and emotional disorders — should be the main target of the training process for medical professionals. The Assistance and Research Center for Medical Residency (Núcleo de Assistência e Pesquisa em Residência Médica — Napreme) was created within Unifesp and effectively implemented in September 1996. In a previous communication^25^ we presented a detailed explanation of the characteristics of the service at the time of its creation as well as the targets and strategies adopted for publicizing its services within the institution.

In addition to psychological and psychiatric healthcare, Napreme has developed several actions aimed at preventing emotional disturbances and professional dysfunctions among trainees. For example, Napreme has been participating in a welcome program for medical and nursing residents by giving lectures on the stress experienced during residency. Group meetings with the residents have provided in-depth discussion of the stressful nature of the residency program and are aimed at working on the resident's expectations, desires and fears regarding the training program. At these meetings, a detailed account is given of Napreme's services, which the residents are encouraged to seek whenever any problems arise. Emphasis is given to ensuring full confidentiality, and to the fact that Napreme is independent of the training assessment system.

Another activity carried out by Napreme is research. A study performed by Obara,^26^ using the Beck Depression Inventory (BDI) and including 75 first-year residents in different clinical and surgical specialties deserves mention. In this study, the prevalence of depressive symptoms was 24% (BDI > 14), thus showing a much higher percentage than in other studies using the same methodology.^10,14^ In another study in association with Napreme, Franco^27^ also used the BDI to evaluate the prevalence of depressive symptoms among 68 nursing residents, finding a rate of 18.6%. At present, the authors of the present study are now in the process of concluding a study on the quality of life and wellbeing of 140 medical residents.

Napreme is located two blocks from the University's main buildings, alongside the student health service (Serviço de Saúde do Corpo Discente, SSCD), which offers medical and dental assistance to the entire student body at Unifesp. Napreme's medical team consists of two psychologists and one psychiatrist, whose services are offered to outpatients in the following fields: psychiatric treatment, individual brief psychotherapy, counseling and psychological orientation.

It should be mentioned that, due to spontaneous demand within the university, it was decided from the outset that healthcare services would be provided not only to residents, but to all students on *stricto sensu* and *lato sensu* postgraduate courses. In the literature available, stress levels are significantly higher among postgraduate students than among medical students or residents.^28^ It has also been shown that physicians taking postgraduate courses seem to belong to a subgroup that is under greater stress than for any other postgraduate students.^29^ The stress experienced by postgraduate students is basically the result of: 1) difficulties in personal relationships with advisors, professors or colleagues; 2) academic activities (classes and scientific production); 3) being pressed for time or facing deadlines; and 4) financial and professional worries.^30^ Regardless of the type of difficulties, the postgraduate students seen at Napreme considered that all the support received was invaluable, in relation to advisors, institutions or professional problems.^31^

Only a few structured services give assistance to this type of population.^22^ Since Napreme was a pioneer in Brazil, its main concern was to create a research database to provide initial guidance for the team, with regard to identifying the trainees who were seeking help, and what their problems were. The present study thus had the aim of describing the clinical and demographic profile of the users of the mental health care services at a public university in Brazil.

## METHODS

This was a retrospective study covering the period from December 1, 1996, to November 30, 2002. During this period, the demographic and clinical variables of each new case attended at Napreme were systematically recorded. The sample consisted of the 233 trainees (medical and nursing residents, and postgraduate students at specialization, master's or doctoral levels) registered during the abovementioned period. The trainees came to the service in three different ways: spontaneous visit, referrals from their supervisors, or referrals from SSCD physicians.

All of the healthcare provided required the filling out of a semi-structured registration form, which was subsequently standardized to build the service's database. The records contained the patient's demographic data and information on their professional training and psychiatric and personal background, such as: gender; age; marital status; how the trainee found out about the service; who referred the trainee; the type of academic relationship with the university; the area of specialization being studied; profession; site of the training program; place of birth; which school the trainee graduated from; home address; clinical history; recent use of medication; habits related to smoking, alcohol and drugs; suicidal ideas; sleep and feeding disorders; previous psychological or psychiatric treatment; family psychiatric background; main diagnosis (International Statistical Classification of Disease and Related Health Problems, 10th Revision, ICD-10);^32^ medication prescribed; results of treatment; and leave of absence required from activities.

The data was recorded in a structured database, using the Statistical Package for Social Sciences (SPSS) version 8.0 software. Seven different psychologists or psychiatrists worked in Napreme during the study period. At the time of each trainee's first consultation, the psychologist or psychiatrist was asked to fill out a semi-structured form, which was then reviewed by the Napreme coordinator.

The descriptive analysis was performed by a direct comparison of the rates, distributed into different categories of variables such as sociodemographic characteristics, demand for the service, or adherence to the treatment. Contingency tables were developed and Pearson's chi-squared test was applied to compare the trainees according to their academic relationship with the university, namely, medical and nursing residents, or postgraduate students at specialization, master's or doctoral levels.

## RESULTS

### Demographics

The average age of the trainees was 27 (SD = 4) years, ranging from 21 to 48 years. The nursing residents were the youngest patients treated at the service, with an average age of 24 years. There was a higher frequency of female patients (79.4%) than of male patients, but upon distributing the groups according to their university qualification, the population of female patients accounted for 67.9% of the medical residents, 97.5% of nursing residents, 83.7% of the postgraduate specialization students, 81.7% of the master's students and 71.4% of doctoral students (Table 1). Thus, while 32.1% of the residents were male patients, only 15.8% of the remaining trainees were male (c^2^ = 7.15; df = 1; p < 0.01).

**Table 1:** Distribution of sociodemographic characteristics according to the trainees’ academic relationship with the university (Universidade Federal de São Paulo, Unifesp)

		Medical resident		Postgraduate specialization		Master's student		Doctoral student		Nursing resident		Total	
		n	%	n	%	n	%	n	%	n	%	n	%
Gender*	Male	18	32.1	9	16.3	11	18.3	8	28.6	2	2.5	48	20.6
	Female	38	67.9	39	83.7	49	81.7	20	71.4	39	97.5	185	79.4
Status ^NS^	Single	46	82.1	43	89.6	45	75.0	18	64.3	39	97.5	191	82.0
	Not single	10	17.9	5	10.4	15	25.0	10	35.7	2	2.5	42	18.0
Graduated at ^NS^	Unifesp	20	39.2	5	10.4	14	26.9	6	25.0	8	22.2	53	22.7
	Another university	36	60.8	43	89.6	46	73.1	22	75.0	33	77.8	180	77.3
Living with ^NS^	family	16	30.2	22	57.1	26	46.4	14	50.0	9	23.7	87	37.3
	other situations (alone, friends)	40	69.8	26	42.9	34	53.6	14	50.0	32	76.3	146	62.7
**Total**		**56**	**24.0%**	**48**	**20.6%**	**60**	**25.8%**	**28**	**12.0%**	**41**	**17.6%**	**233**	**100.0**

*NS: differences are not significant;*

* *p < 0.05.*

Eighty-two percent of the patients treated were single, whereas there were fewer single patients among the master's and doctoral students. There were smaller numbers of medical and nursing residents living with their families than for the trainees in other groups. A significant number of trainees had not graduated at Unifesp (77.3%).

### Professional qualifications

The population consisted of physicians (31.3%), nurses (20.6%), biomedics (12.9%) and other health professionals (35.2%).

The medical and nursing residents and postgraduate students at specialization, master's and doctoral levels who were seeking treatment participated as staff members in different departments and sectors of the university. Of these, the most important ones were: Pediatrics and Nephrology, with 6.4% of the cases treated; Speech Therapy with 6%; Nutrition with 4.3%; Pharmacology and Molecular Biology with 5.2% each; and Ophthalmology and Neurology with 3.9% each. A total of 51 departments and sectors at the University used the services provided by Napreme.

Most of the patients were trainees from the clinical sectors (52.4%) and laboratory researchers (30.5%). The surgical sector contributed with 9.9% of the cases whereas the field of diagnostic techniques and methods accounted for 3.4%.

### Demand for the service

Although patients usually sought help spontaneously (in 70.8% of the cases), 29.9% of the cases came via referrals. Fifty-eight cases were treated during the first two-year period covering the month of December 1996 and 1997-1998. In the second two-year period (1999-2000) there were 100 new cases and in the third two-year period (2001-2002) another 75 new cases were recorded. The distribution of the demand for primary mental healthcare was characterized by a major occur-rence during the period from May to September, coinciding with the middle of the annual training cycle, which runs from February of one year to January of the next year.

Regardless of the type of academic relationship with the University, either as medical and nursing residents, or as postgraduate students at specialization, master's or doctoral level, there was a higher frequency of patients seeking treatment in the first year of the training program (Table 2). There was also a reduction in the need to search for help as the training program progressed: 63.1% of the patients seeking treatment were in their first year, 22.7% in their second year, 7.7% in their third year, 1.3% in their fourth year and 0.9% were in their fifth year.

**Table 2:** Distribution of first consultation at the service, according to the trainees’ academic relationship with the university (Universidade Federal de São Paulo, Unifesp)

		Medical resident		Postgraduate specialization		Master's student		Doctoral student		Nursing resident		Total	
		n	%	n	%	n	%	n	%	n	%	n	%
Year of training	First year	32	57.1	38	71.4	34	61.8	13	46.4	30	76.9	147	63.1
Program^NS^	Other years	24	42.9	10	28.6	26	38.2	15	53.6	11	23.1	86	46.9
**Total**		**56**	**24.0%**	**48**	**20.6%**	**60**	**25.8%**	**28**	**12.0%**	**41**	**17.6%**	**233**	**100.0**

*NS: differences are not significant.*

### Adherence to treatment

The majority of patients adhered to their treatment (67.4% of the cases). At the end of the study period (November 2002), 13.3% of the total population studied were being regularly treated at the service, 27.9% had been discharged from treatment, 32.6% had discontinued their treatment, 21.9% had requested a referral for psychotherapy or psychiatric treatment elsewhere, and the data was incomplete for 4.3% of the cases. Among the cases that came to the service after being referred by supervisors, a much higher proportion (61.1%) dropped out from the treatment (χ^2^= 4.48; df = 1; p < 0.05).

### Clinical characteristics

When the patients came to the service, 12.9% reported some kind of clinical disease; 13.3% mentioned having used some kind of non-psychotropic medication during the last month; and 13.3% stated that they had taken some kind of psychotropic drugs, with benzodiazepines being used by 7.7% of these patients. In 73.4% of the cases, no reference was made to the use of medication.

A little more than half (52.4%) of this population reported having refrained from the use of alcoholic beverages, whereas 31.3% were “social drinkers” only. Of these patients, 4.7% were not only “social drinkers”, and 2.1% believed they had increased the use of alcohol over the last month (Table 3). In the group of medical residents, there were clearly fewer individuals who refrained from the use of alcoholic beverages (χ^2^ = 4.46; df = 1; p < 0.05).

**Table 3:** Distribution of clinical characteristics according to the trainees’ academic relationship with the university (Universidade Federal de São Paulo, Unifesp)

		Medical resident		Postgraduate specialization		Master's student		Doctoral student		Nursing resident		Total	
		n	%	n	%	n	%	n	%	n	%	n	%
Alcohol use*	Yes	23	45.1	26	54.2	28	52.8	17	65.4	28	75.7	122	52.4
	No	33	54.9	22	45.8	32	47.2	11	34.6	13	24.3	111	47.7
Sleep disorder*	Yes	31	60.8	14	29.2	28	52.8	10	38.5	18	48.6	101	43.3
	No	25	39.2	34	70.8	32	47.8	18	51.5	23	51.4	132	56.7
Leave of absence required**	Yes	11	20.8	3	6.3	1	1.7	3	10.7	1	2.6	19	8.2
	No	45	79.2	45	93.7	59	98.3	25	89.3	40	97.4	214	91.8
Suicidal ideas^NS^	Yes	13	25.0	10	20.8	14	25.5	5	17.9	10	26.3	52	22.3
	No	43	75.0	38	79.2	36	74.5	23	82.1	31	73.7	181	77.7
**Total**		**56**	**24.0%**	**48**	**20.6%**	**60**	**25.8%**	**28**	**12.0%**	**41**	**17.6%**	**233**	**100.0**

*NS: differences are not significant;*

* *p < 0.05,*

** *p < 0.001.*

Of the patients who were attended, 11.6% had already thought of committing suicide, 3.4% had previously attempted suicide, 22.3% were having suicidal ideas at the time of the first interview (Table 3), and 0.4% not only had already attempted suicide previously, but were also having suicidal ideas at that very moment.

Sleeping disorders proved to be quite frequent at the time of the first visit (Table 3). These were found in 43.3% of the patients, of whom 25.3% were suffering from insomnia, 8.6% complained of hypersomnia and 9.4% had sleeping disturbances. The medical residents showed a higher incidence of sleeping disorders than among the other trainees (χ^2^ = 4.50; df = 1; p < 0.05; Table 3).

Changes in appetite were reported in 42.1% of the patients, of whom 17.2% had less appetite and 24.9% had an increase in appetite.

A little over half of the individuals who sought Napreme (54.1%) had never previously looked for any type of psychological or psychiatric help. Among those who had previously been treated, 22.3% had received psychotherapy; 9.4% had had psychiatric treatment; 3% had had psychiatric and psychotherapy treatment and 4.7% mentioned other kinds of treatment.

With regard to diagnoses, it should be noted that 30% of the cases it was not possible to meet the ICD-10 criteira^32^ for diagnosis. 13.3% had adjustment disorders (F43.2); 11.2% had episodes of light depression (F32.0); 8.2% had episodes of moderate depression (F32.1); 7.3% had personality disorders (F60); 5.6% had mixed anxiety and depression disorders (F41.2); 5.2% had generalized anxiety disorders (F41.1); and 19.2% were diagnosed as other minor disorders.

During the course of treatment, psycho-tropic drugs were not prescribed in 70.8% of the cases. In 4.7% of the cases, anxiolytic drugs were prescribed; 11.2% were given antidepressant drugs; 0.4% were given mood stabilizers; and 8.6% were given more than one type of psychotropic drug.

Another difference observed when comparing the medical residents with the other users of the Napreme services was in relation to the need for temporary permits for sick leave. The medical residents needed to take leave of absence from work due to illness in 20.8% of the cases, whereas for the other trainees this was only required in 4.7% of the cases. This difference was found to be statistically significant (χ^2^ = 13.4; df = 1; p < 0.001; Table 3). Of the 11 medical residents who took sick leave, three ended up abandoning their residency program.

## DISCUSSION

After six years of activity, it is now possible to present a profile of Napreme users. After the first two years of activities, while this new service was still being structured, it was already achieving some stability in the numbers of patients seeking help, at a level of approximately 50 new cases per year. A specific monthly demand pattern was also noted. During the first and last months of the year there was a drop in the demand for treatment at the service, which then increased and reached its highest concentration during the period from May to September. With regard to the medical residents, this increase in demand has been explained in studies that have shown a correlation between the first year of training and a sharp increase in stress.^24^ It was in the first year that the greatest demand occurred, and this lends further support to other findings in the literature that indicate that the first year is the most stressful.^3,5,8,9,16^

This demand pattern for psychological help was also found among the nursing residents and students on other postgraduate courses. Thus, it would be reasonable to suggest that the stress pattern among medical residents could be the general pattern felt by the entire postgraduate student body, particularly during the first year of a specialized healthcare training program. In any event, such relevant data should be passed on to the directors of healthcare training programs, with the recommendation that careful attention should be given to trainees during their first year, especially through ensuring constant supervision.

The prevalence of female patients among the population treated at Napreme is a sign of the growing participation of women in residency programs and postgraduate work. Women are also inclined to be more susceptible to depressive symptoms and emotional disorders during training programs^7,11,16^ and, when faced with emotional suffering, tend to seek more help than men do.^33^

The fact that sleep disorders were more prevalent among medical residents than in other groups is probably due to sleep deprivation, which is a significant cause for concern in the training program. This also reinforces the movement in favor of discontinuing “36 x 12” shifts, or schemes of 36 hours of work followed by 12 hours of rest. It furthermore contributes towards advocating a policy for setting a limit to medical residents’ workload. In Brazil, a law regulating the weekly workload to 60 hours came into force in 1981.^34^

Another important finding from the present study relates to the greater need among medical residents to take time off on sick leave than among other groups. This appears to be consistent with the intense activity required in attending to patients and the more stringent commitments, shifts and schedules that characterize the medical residency training program.

There were fewer medical residents than other trainees treated at Napreme who refrained from the use of alcohol. This is probably because there was a significantly greater number of male patients in the group of medical residents than in the other trainee subgroups. Nonetheless, after adjusting this incidence for gender, its significance remained unaltered. In another study with similar findings regarding the statistical correlation between emotional problems and the use of alcohol among medical residents, the significance remained unaltered when the incidence of personal problems was adjusted for gender, using variance analysis.^35^

In the cases in which a formal psychiatric diagnosis was established, there was a higher prevalence of anxiety-depressive disorders. This was also found in a survey within a similar service at the University of California, Los Angeles.^23^

In 30% of the cases we failed to meet the ICD-10 criteria^32^ for formulating a psychiatric diagnosis. This finding was probably biased, because Napreme usually adopts the policy of distinguishing between the degrees of psychological suffering, which has often prevented the formulation of a more precise diagnosis according to the ICD-10 criteria, for example in the diagnosis of adaptation disorders. In a certain number of cases, a more appropriate diagnosis would probably have been “transi-tory self-limited deadaptation”. It should be kept in mind that most of the problems faced during the training course can often be overcome with the help of a “mentor”: someone who is more experienced or better adapted, and who knows how to be sympathetic and offer support to other people.^24,31^

In addition to these situations that could be classified as “transitory deadaptation”, there were other very serious situations, in which the classification in and by itself failed to convey the full drama that was taking place. Three of the most serious cases were of female residents who, after being on sick leave for a certain period, finally abandoned the residency training program, due to psychopathological problems. One of these patients, besides having a severe personality disorder, was suffering from heavy abuse of illegal drugs. Despite the significant support provided by colleagues and supervisors, the patient failed to comply with the treatment and abandoned the university without ever responding to our attempts to contact her. The second case was of a resident with a well structured family and financial resources, who had been under psychiatric treatment and psychotherapy ever since she had been an undergraduate at medical school. The patient's serious clinical condition was diagnosed as an obsessive-compulsive disorder, and this prevented her from continuing with the training course, despite the several attempts that she made to return. The third case was of a female resident whose paranoid personality disorder caused serious conflicts with her colleagues, supervisors, patients and family members. The situation worsened to such a point that the pressure of the hostility she had created became unbearable and she finally abandoned the training program. Although the patient's compliance with treatment had been quite irregular, she managed to create friendly ties with one of the service's psychologists and, before leaving the university, she asked this psychologist to recommend another psychologist who could help her to continue with the treatment.

The seriousness of the cases attended is also clearly evident in relation to suicide. In the present study, 22.3% of the trainees had what they described as suicidal thoughts. It is a well-known fact that self-destructiveness is highly prevalent among healthcare professionals, and particularly among physicians. The creation of Napreme was highly influenced by the suicide of four young physicians. This caused a severe impact on the university, especially because one of these physicians died by jumping from the 10^th^ floor of the hospital during a 24-hour shift. The other two were cared for and admitted to the university hospital, where they eventually died. The impact of these events not only contributed towards the creation of the service, but also towards increased sensitivity regarding university issues and the mental health of professionals on training programs. In this sense, more extensive, in-depth discussions on these matters have gradually been made possible. It has also become easier to develop studies in this area, for example a study on depression that was carried out among medical and nursing residents.^26,27^ It should also be noted that this type of service is officially recommended in the United States for all schools offering postgraduate courses in the field of medicine.^12^

The acceptance and institutional credibility of Napreme's services appears to be very positive. This is demonstrated by the spontaneous demand for such services and also by the fact that, in over half of the cases, the users themselves have recommended the service. The credibility that induces patients to spontaneously seek help from the service seems to be related to the lower rate of discontinuation of treatment, when patient spontaneously come to the service for help.

Napreme was created to provide services for young academically productive professionals. Most of these individuals are directly responsible for hospital care, for the making of life and death decisions regarding patients and for teaching activities within the university. The service is not only concerned with the needs of these patients, but also with those of the university it belongs to. The situations to be dealt with range from psychosis to personality disorders, from deadaptation to the working environment and family conflicts. For this, Napreme functions in an extremely flexible manner encompassing different actions in different situations, with contingencies for variability in demand. At certain periods of the year, demand can increase fivefold in relation to other periods.

## CONCLUSION

Among the different groups of trainees treated there were more similarities than differences. This shows that the concentration of psychological/psychiatric care within a single service is appropriate for this population.

The creation of services such as Napreme must be encouraged at all institutions offering professional training in the medical field, since such services can deal with many of the problems that cause an impact on both the institution and its trainees.
